# Left ventricle chest compression improves ETCO_2_, blood pressure, and cerebral blood velocity in a swine model of cardiac arrest and cardiopulmonary resuscitation

**DOI:** 10.1016/j.resplu.2022.100326

**Published:** 2022-11-14

**Authors:** Rory A. Marshall, Jude S. Morton, Adam M.S. Luchkanych, Yehia El Karsh, Zeyad El Karsh, Cameron Morse, Corey R. Tomczak, Brian E. Grunau, T. Dylan Olver

**Affiliations:** a Biomedical Sciences, Western College of Veterinary Medicine, University of Saskatchewan, Saskatoon, SK, Canada; b British Columbia Emergency Health Services, Vancouver, BC, Canada; c Health and Exercise Sciences, University of British Columbia Okanagan, Kelowna, BC, Canada; d College of Kinesiology, University of Saskatchewan, Saskatoon, SK, Canada; e Departments of Emergency Medicine, St. Paul’s and the University of British Columbia, Vancouver, BC, Canada

**Keywords:** Left Ventricle Chest Compressions, Cerebral Blood Flow, Chest Compressions, Basic Life Support, CPR, Cardiac Arrest

## Abstract

**Introduction:**

During cardiopulmonary resuscitation (CPR), high quality chest compressions are critical to organ perfusion, especially the brain. Yet, the optimal location for chest compressions is unclear. It was hypothesized that compared with the standard chest compression (SCC) location, left ventricle chest compressions (LVCCs) would result in greater ETCO_2_, blood pressure (BP), and cerebral blood velocity (CBV) during CPR in swine.

**Methods:**

Female Landrace swine (N = 32; 35 ± 2 kg) underwent two mins of untreated asphyxiated cardiac arrest (CA). Thereafter, swine were treated with three 2-min cycles of either SCC or LVCC mechanical basic life support CPR (LUCAS 3). ETCO_2_ (in-line sampling), BP (arterial catheter line), and CBV (transcranial Doppler) were measured during the pre-CA, untreated-CA, and CPR-treated phases.

**Results:**

ETCO_2_, BP, and CBV were similar between groups at pre- and during untreated-CA (P ≥ 0.188). During CPR, ETCO_2_ (36 ± 6 versus 24 ± 10 mmHg, P < 0.001), mean arterial BP (MAP; 49 ± 9 versus 37 ± 9 mmHg, P = 0.002), and CBV (11 ± 5 versus 5 ± 2 cm/s, P < 0.001) were significantly greater in the LVCC versus SCC group. Moreover, a greater proportion of animals obtained targets for ETCO_2_ (ETCO_2_ ≥ 20 mmHg; 52 % (17/33) versus 100 % (32/32), P < 0.001) and diastolic BP (DBP ≥ 25 mmHg; 82 % (33/40) versus 97 % (48/49), P = 0.020) in the LVCC versus SCC group.

**Conclusion:**

Indicators of cardiac output, BP, and cerebral perfusion during CPR were greatest in the LVCC group, suggesting the quality of chest compressions during BLS CPR may be improved by performing compressions over the left ventricle compared to the centre of the chest.

## Introduction

Cardiac arrest (CA) occurs over 500,000 times annually in the United States.^1^ Reduced cerebral perfusion during CA and cardiopulmonary resuscitation (CPR) is implicated in poor survival rates (<10 %)2., 3, 4, 5 and post-arrest neurological deficits.^6^ Improvements to CPR that increase cerebral blood flow may lead to profound life saving benefits.

Current CPR guidelines define the surface landmark of standard chest compression (SCC) as the centre of the chest on the lower half of the sternum.^7^ This SCC location overlies the upper third of the heart, which encompasses the aortic root, ascending aorta, left ventricular outflow tract, or atria in over 80 % of patients.^8^, ^9^, ^10^, ^11^, ^12^, ^13^, ^14^, ^15^, ^16^ Compressing these structures does not compress the greatest volume of the heart and may increase cardiac outflow resistance.^13^, ^14^, ^17^

Left ventricle chest compression (LVCC) during CPR may offer hemodynamic benefits compared to SCC.^18^, ^19^ LVCC is performed by identifying the location overlying the left ventricle on the anterior chest wall and delivering external chest compressions at that site. Specifically, LVCC targets a larger volume of the heart and avoids compression of outflow vessels, thereby increasing stroke volume and potentially decreasing outflow resistance. In swine models of ventricular fibrilation and traumatic CA, LVCC has been reported to improve systemic hemodynamics and return of spontaneous circulation (ROSC).^19^, ^20^, ^21^, ^22^ Whether the benefits of LVCC extend to the cerebral circulation remains unknown. Using a swine model of asphyxiated CA, the purpose of this study was to compare standard indicators of hemodynamic status and cerebral blood flow between SCC and LVCC during basic life support (BLS; chest compressions and ventilations only) CPR. It was hypothesized that compared to SCC, LVCC would result in greater ETCO_2_ (established indicator of cardiac output^23^), blood pressure (BP), and cerebral blood velocity (CBV; validated indicator of cerebral blood flow^24^, 25., 26, 27) during BLS CPR.

## Methods

### Study design

This prospective interventional trial was approved by the University of Saskatchewan Animal Research Ethics Board (#20200042). Briefly, animals underwent asphyxiated CA and received either SCC or LVCC BLS CPR while hemodynamics (ETCO_2_, BP and CBV) were monitored.

### Animal preparation

Following acclimatization and a 12-hour overnight fast, female Landrace swine (N = 32; 35 ± 2 kg; Prairie Swine Center, CAN) were sedated with an intramuscular injection of 20–30 mg/kg of ketamine (Vetoquinol, CAN). Swine were intubated and mechanically ventilated (10 ml/kg tidal volume; Excel 210 SE, 7900 SmartVent, Datex Ohmeda, FIN). Anesthesia depth was maintained with 1–3 % isoflurane (Baxter, CAN). Body temperature was maintained at ∼ 38 °C (rectal thermometer). An intravenous (IV) catheter was inserted into an auricular vein for fluid maintenance (10 ml/kg/hr normal saline) and medication delivery.

Animals were placed supine in a V-shaped holder with the limbs and head secured to prevent displacement during CPR.^19^, ^20^, ^21^, ^22^, ^28^ A gas analyzer (ADI, USA) sampling line was connected to the ventilatory circuit and the ventilation rate was adjusted to maintain an ETCO_2_ range of 35–40 mmHg.^29^, ^30^ Continuous heart rate and cardiac activity were monitored by electrocardiogram (ECG) using a standard 3-lead limb system (ADI).^19^, ^20^, ^21^, ^22^ Beat-by-beat BP was measured from a femoral artery catheter (BD, USA) connected to a pressure transducer^6^, ^30^, ^31^ (DELTRAN II, Utah Medical Products, USA). To prevent clotting, an initial IV 300 IU/kg bolus of heparin was administered with an additional 75 IU/kg bolus administered every hour.^19^, ^21^

**Fig. 1:**
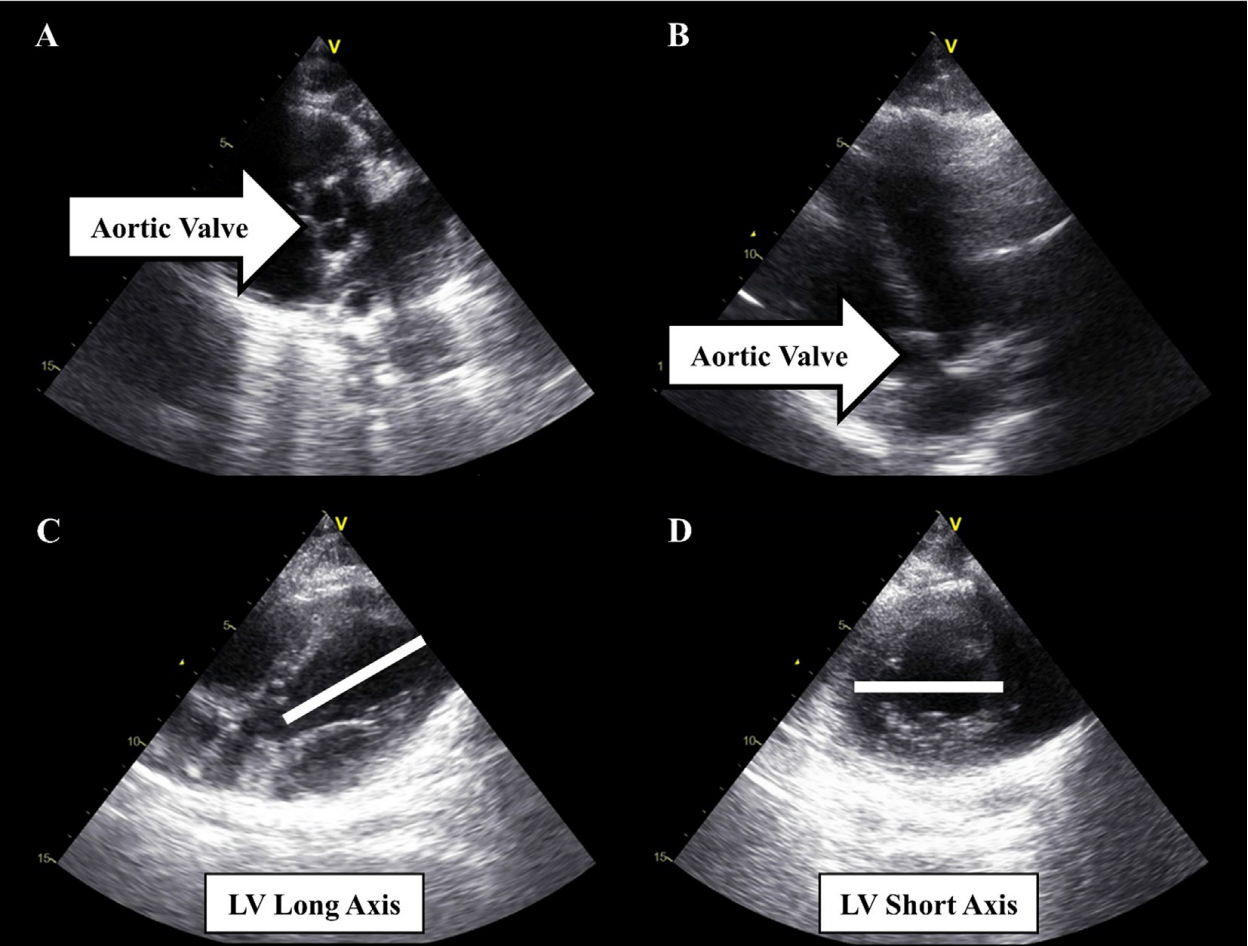
Transthoracic echocardiography 2-dimensional images of the aortic valve **(A & B)** to guide surface markings for the standard chest compression (SCC) location. Left ventricular long **(C)** and short **(D)** axes used to guide surface markings for the left ventricle chest compression (LVCC) location. The white lines in **C** and **D** indicate the corresponding axes.

Following instrumentation, transthoracic echocardiography (GE Vivid I, GE 3Sc-Rs Probe; CCE Medical, CAN) was used to locate and mark the surface site for either SCC (sternal midline at the level of the aortic root; Fig. 1A and 1B)^8^, ^10^, ^19^ or LVCC (intersection of the parasternal long and short axes of the left ventricle; Fig. 1C and 1D).^19^, ^20^, ^21^, ^22^ Although the level of the aortic root is not indicative of all clinical SCC locations, this normalized location^19^, ^20^, ^21^, ^22^ was selected as the experimental control as it encapsulates the majority of clinical chest compression sites.^8^, ^9^, ^10^, ^11^, ^12^, ^13^, ^14^, ^15^, ^16^ A transcranial Doppler (TCD, Multigon Industrial Inc., USA) signal was then obtained to assess indices of cerebral blood flow.^24^, 25., 32., 33 Briefly, a pencil probe (2 MHz) was positioned superficially over the right transorbital window to obtain middle cerebral artery CBV.^24^, 25., 26, 27, 31, 34 Time-aligned indices of systemic hemodynamic status (ETCO_2_, heart rate, BP) and CBV were recorded using a digital-analog converter (Powerlab; ADI) interfaced with a data collection software program (LabChart 8, ADI) during baseline, CA and CPR.

### Experimental protocol

Following a 60-min stabilization period,^35^ a mechanical chest compression device (LUCAS 3, Stryker, SWE) was positioned and secured over the previously assigned SCC or LVCC surface landmark.^19^, ^20^, ^21^, ^22^ Once fully instrumented, pre-CA baseline data were collected for 2 mins. Asphyxiated CA was induced by cessation of mechanical ventilation and occluding the endotracheal tube. Animals were given a 5–20 mg/kg bolus of propofol to prevent gasping (Baxter).^36^, ^37^ CA was confirmed by loss of cardiac motion (echocardiography) and total absence of a carotid Doppler pulse (long axis view; GE Vivid I, GE 9L Probe; CCE Medical).^38^, ^39^ Following CA confirmation, 2 mins of untreated CA was allotted prior to beginning BLS CPR.

During BLS CPR, mechanical ventilation was resumed and kept constant on 100 % oxygen.^19^, ^20^, ^21^, ^22^ Consistent with the American Heart Association (AHA) 2020 CPR guidelines, the chest compression rate was 100/min, compression depth was 5 cm, and full thoracic recoil ensured.^7^ BLS CPR was performed for three 2-min rounds separated by 10-sec circulation checks.^7^ Hemodynamic data during BLS CPR were grouped and averaged for each 2-min round. If ROSC was achieved during a circulation check, the protocol was terminated. Successful ROSC was defined as the presence of a carotid Doppler pulse^38^, ^39^ and sustained regular electrical and mechanical cardiac activity producing a systolic BP (SBP) ≥ 60 mmHg without intervention for at least 1-min.^19^, ^20^, ^21^, ^22^ If an animal did not achieve ROSC for a full min then the protocol was resumed at the point at which it was paused.^19^, ^20^, ^21^, ^22^ Animals that achieved ROSC were kept on mechanical ventilation (1–3 % isoflurane) without additional intervention for up to 10 mins prior to euthanization.^19^, ^20^, ^21^, ^22^ Following euthanasia (exsanguination), post-mortem dissection was conducted to assess for injuries.

### Statistics

Owing to the blatant difference in chest compression locations, experimenters could not be blinded during CPR administration. However, data analysis was completed by a researcher blinded to experimental condition. Data during the entire 2 mins of baseline, untreated CA and each round of BLS CPR were binned independently and averaged for each period. Erroneous waveforms that exceeded 1 standard deviation from the mean were removed. Statistical analyses were completed using SigmaPlot 14.0 (SysStat, USA). Sample size was estimated using ETCO_2_ data from previous SCC versus LVCC swine work.^19^ This prior report demonstrated an 11 mmHg difference (standard deviation approximating 50 % of group means) in ETCO_2_ favouring LVCC over SCC.^19^ We computed that a minimum n = 10 would be required to detect an 11 mmHg difference in ETCO_2_ between LVCC and SCC, assuming two-tailed significance of 0.05, 80 % power, and an ETCO_2_ response variance approximating 70 % of the mean difference (*i.e.*, 8 mmHg). Employing a modified version of the ARRIVE guidelines’ simple randomization procedure,^40^ the first animal was randomized (SCC or LVCC; Random.org) and thereafter animals were rotated systematically (*i.e.,* SCC, LVCC, repeat) to achieve the necessary sample size. Potential nuisance variables were balanced between groups with strict standardized animal care, housing, feeding interaction and handling procedures, using the same investigators for animal instrumentation and experimentation, and using animals of the same sex and similar mass.

Indices of hemodynamic status were compared using a group (SCC versus LVCC) × time (baseline, CA, BLS CPR round 1, 2, and 3) mixed model ANOVA. *A priori* between group pairwise comparisons at baseline, CA, and round 1, round 2 and round 3 of BLS CPR were completed using a post hoc Tukey’s test. Within group differences were not explored. Grouped data are presented as mean with individual data points. Cohen’s d effect size analysis (small effect ≥ 0.20; medium effect ≥ 0.50; large effect ≥ 0.80) were calculated to compare the magnitude and directional effects of LVCC versus SCC on systemic and cerebral hemodynamic status during each round of CPR.^41^ Fisher’s exact tests were used to assess for differences between chest compression location and the number of animals that reached AHA targets for ETCO_2_ (≥20 mmHg)^7^ and DBP (≥25 mmHg)^1^ in each round and overall BLS CPR. Fisher’s exact tests were also used to assess for differences between chest compression location and the number of animals that achieved ROSC, as well as the number of animals that incurred injury. All Fisher’s exact data are presented as percent achieved [achieved/n]. Significance for all tests was considered at P < 0.05.

## Results

To ensure statistical power for all measures and to account for incomplete data, 32 animals underwent the CPR protocol (SCC n = 14, LVCC n = 18). There was incomplete data for three animals in the LVCC group (ROSC). Further, BP data was missing from n = 1 in the SCC group (accidental catheter removal), ETCO_2_ data was missing from n = 3 in the SCC and n = 4 in the LVCC group (equipment malfunction), and CBV data was missing from n = 2 in each group (loss of signal). After the initial 20 experiments, LVCC group had a greater number of incomplete data sets; thus, the alternation order was revised to attain necessary sample sizes (1. SCC, 2. LVCC. 3. LVCC, repeat). Final samples sizes are listed in each figure legend and individual data points are shown.

### Cerebral blood velocity

**Fig. 2:**
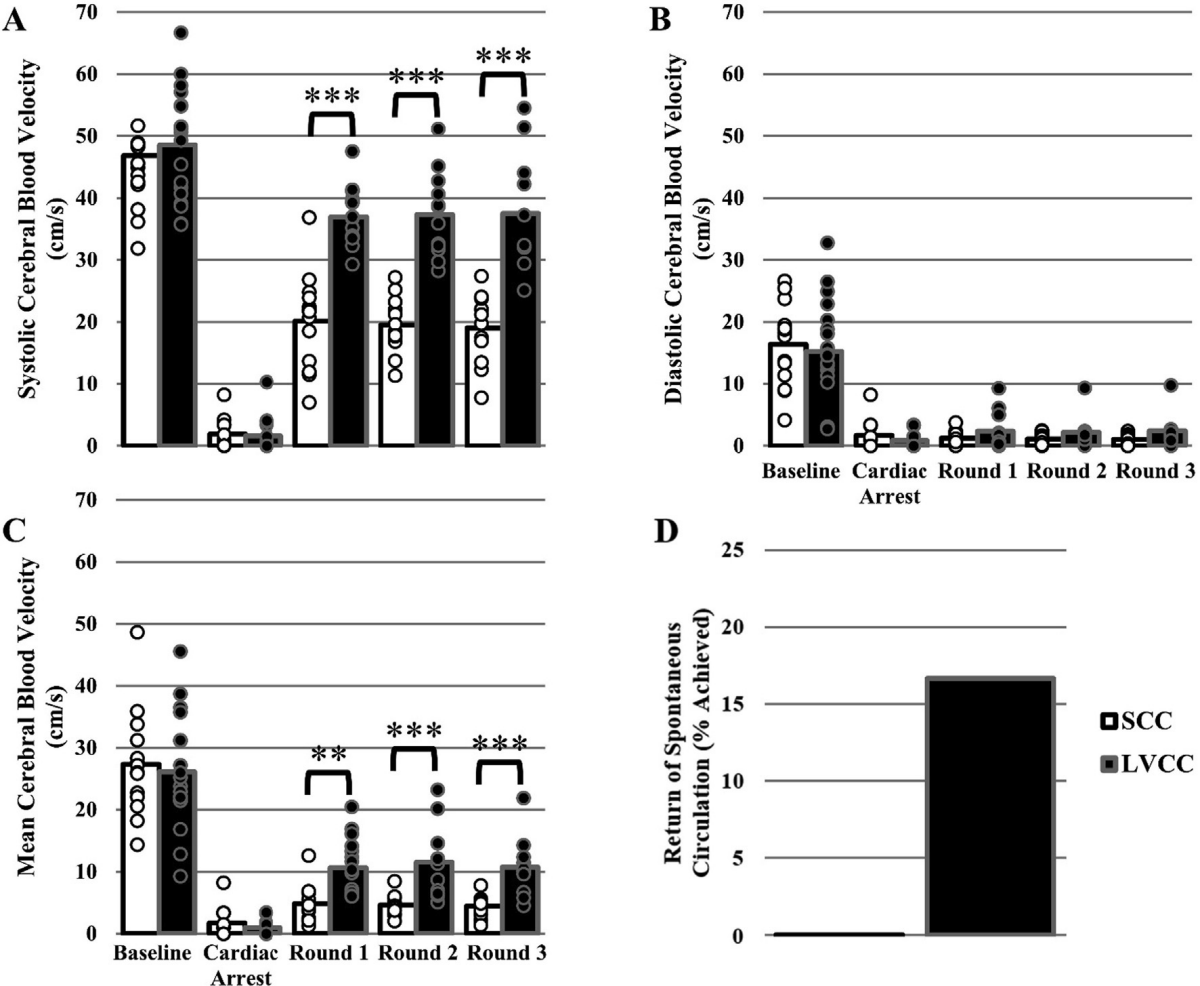
Average systolic cerebral blood velocity (SCBV; Panel A), diastolic cerebral blood velocity (DCBV; Panel B) and mean cerebral blood velocity (MCBV; Panel C) values for standard chest compression (SCC; white fill bars) and left ventricle chest compression (LVCC; black fill bars) groups during baseline, cardiac arrest (CA), basic life support (BLS) cardiopulmonary resuscitation (CPR) round 1 (SCC n = 12, LVCC n = 16), CPR round 2 (SCC n = 12, LVCC n = 12), and CPR round 3 (SCC n = 12, LVCC n = 11). Data were analyzed using a mixed model ANOVA. *A priori* between group comparisons at baseline, CA, aand round 1, round 2 and round 3 of BLS CPR were completed using a post hoc Tukey’s test. Significantly greater than SCC **(P < 0.01), ***(P < 0.001). Bars represent mean. Individual data points reflect individual animals. Some individual datapoints may overlap, thus obscuring visualization. The sample sizes listed herein are accurate. Percent of animals that achieved return of spontaneous circulation (ROSC) in the SCC and LVCC groups during CPR (Panel D). Panel D data were analyzed using a Fisher’s exact test.

There was a significant group × time interaction effect (P < 0.001) for systolic CBV values. Subsequent pairwise comparisons (all completed using a post hoc Tukey’s test) reveal groups were similar at baseline and during untreated CA (P ≥ 0.506), but greater in the LVCC versus SCC group in all three rounds of CPR (P < 0.001; Fig. 2A). There was no group × time interaction effect for diastolic CBV values (P = 0.309; Fig. 2B). There was a significant group × time interaction effect (P < 0.001) for mean CBV values. Pairwise comparisons reveal groups were similar at baseline and during untreated CA (P ≥ 0.512), but greater in the LVCC versus SCC group in all three rounds of CPR (P ≤ 0.006; Fig. 2C). The effect size analysis reveals LVCC had a medium-large, positive effect on systolic CBV, diastolic CBV and mean CBV in the LVCC versus SCC group in all three rounds of CPR (Table 1).

**Table 1:** Effect of LVCC on systemic and cerebral hemodynamics.

**Measure**	**Round 1**	**Round 2**	**Round 3**
	**d**	**d**	**d**
**ETCO_2_ (mmHg)**	+0.3	+0.4	+0.5
**SBP (mmHg)**	+0.3	+0.3	+0.3
**DBP (mmHg)**	+0.2	+0.2	+0.2
**MAP (mmHg)**	+0.3	+0.3	+0.3
**PP (mmHg)**	+0.6	+0.5	+0.4
**SCBV (cm/s)**	+0.6	+0.6	+0.7
**DCBV (cm/s)**	+0.6	+0.7	+0.8
**MCBV (cm/s)**	+0.7	+0.9	+0.8

Cohen’s d effect size (d; + indicates a positive effect; small effect ≥ 0.20; medium effect ≥ 0.50; large effect ≥ 0.80) for end-tidal carbon dioxide (ETCO_2_), systolic blood pressure (SBP), diastolic blood pressure (DBP), mean arterial blood pressure (MAP), pulse pressure (PP), systolic cerebral blood velocity (SCBV), diastolic cerebral blood velocity (DCBV), mean cerebral blood velocity (MCBV) in each round of basic life support (BLS) cardiopulmonary resuscitation (CPR).

### Return of spontaneous circulation and injuries

Swine that did not achieve ROSC presented with asystole (n = 29) during all three circulation checks. Swine that did achieve ROSC (n = 3) presented with asystole during untreated CA and/or circulation checks prior to regaining independent circulation. The occurrence of ROSC was not dependent on chest compression location (Fig. 2D; Table 2). Similarly, the occurrence of rib fractures, sternal fractures, liver lacerations, spleen lacerations, or hemothoraces were not dependent on chest compression location (Table 2).

**Table 2:** ROSC and Injury data between groups.

**Event**	**SCC**	**LVCC**	**P**
	% [occurrence/n]	% [occurrence/n]	
**ROSC**	0 % [0/14]	17 % [3/18]	0.238
**Rib Fractures**	100 % [14/14]	100 % [18/18]	1.000
**Sternal Fractures**	0 % [0/14]	0 % [0/18]	1.000
**Liver Lacerations**	0 % [0/14]	0 % [0/18]	1.000
**Spleen Lacerations**	0 % [0/14]	0 % [0/18]	1.000
**Hemothoraces**	21 % [3/14]	28 % [5/18]	1.000

Percent, proportion, and Fisher’s exact values for return of spontaneous circulation (ROSC), rib fractures, sternal fractures, liver lacerations, spleen lacerations, and hemothoraces between standard chest compression (SCC) and left ventricle chest compression (LVCC) groups following basic life support (BLS) cardiopulmonary resuscitation (CPR).

### End-tidal carbon dioxide

**Fig. 3:**
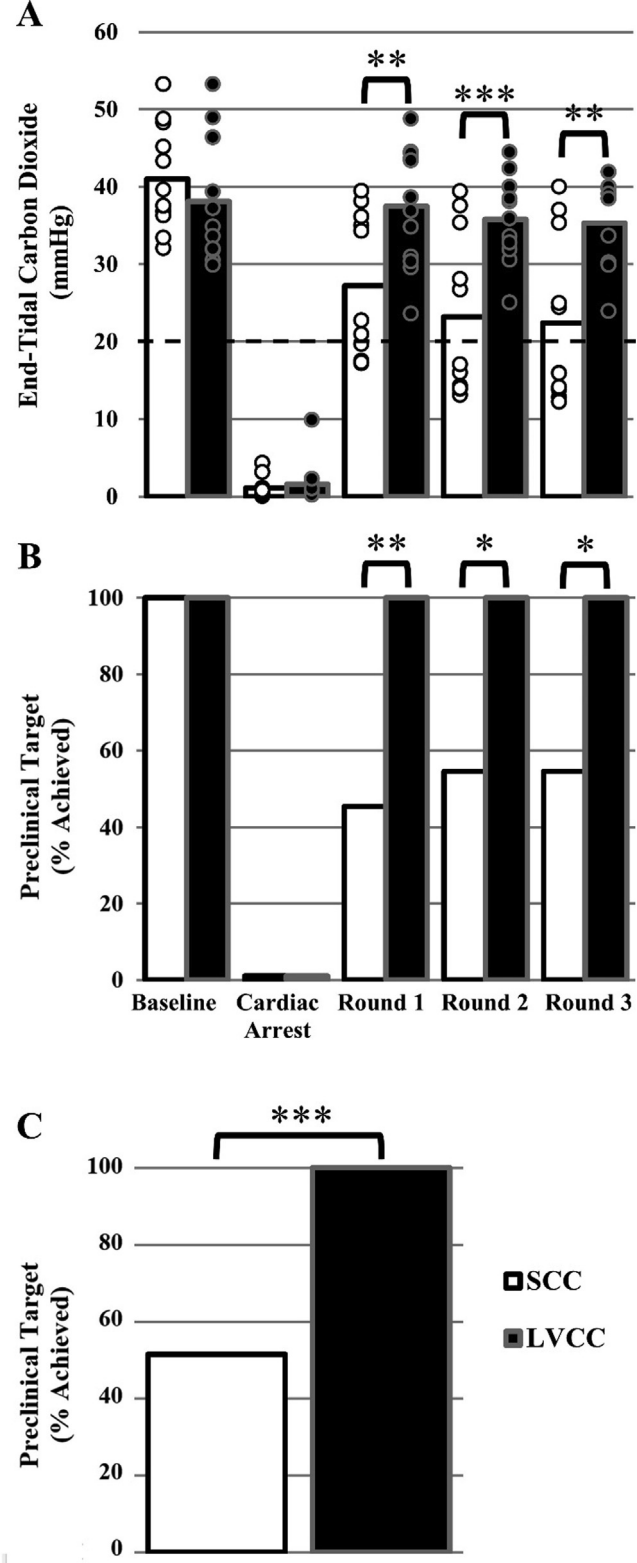
Average end-tidal carbon dioxide (ETCO_2_; Panel A) values for standard chest compression (SCC; white fill bars) and left ventricle chest compression (LVCC; black fill bars) groups during baseline, cardiac arrest (CA), basic life support (BLS) cardiopulmonary resuscitation (CPR) round 1 (SCC n = 11, LVCC n = 12), CPR round 2 (SCC n = 11, LVCC n = 11), and CPR round 3 (SCC n = 11, LVCC n = 9). Data were analyzed using a mixed model ANOVA. *A priori* between group comparisons at baseline, CA, and round 1, round 2 and round 3 of BLS CPR were completed using a post hoc Tukey’s test. Significantly greater than SCC **(P < 0.01), ***(P < 0.001). Bars represent mean. Individual data points reflect individual animals. Some individual datapoints may overlap, thus obscuring visualization. The sample sizes listed herein are accurate. Dashed black line represents preclinical target for ETCO_2_ (≥20 mmHg). Percent of animals that achieved the preclinical target for ETCO_2_ in the SCC and LVCC groups during baseline, CA, and round 1, round 2 and round 3 of CPR **(B)**, and across all three CPR rounds cumulatively **(C)**. Panel **B** and **C** data were analyzed using Fisher’s exact tests. Significant dependencies between chest compression location and achieving the preclinical target by round *(P < 0.05), **(P < 0.01), ***(P < 0.001).

There was a significant group × time interaction effect (P < 0.001) for ETCO_2_ values. Pairwise comparisons reveal groups were similar at baseline and during untreated CA (P ≥ 0.226), but greater in the LVCC versus SCC group in all three rounds of CPR (P ≤ 0.003; Fig. 3A). The effect size analysis reveals LVCC had a small-medium, positive effect on ETCO_2_ throughout CPR (Table 1). Moreover, a greater percent of animals achieved the preclinical target for ETCO_2_ in the LVCC versus SCC group in each round of CPR (P ≤ 0.038; Fig. 3B) as well as across all rounds of CPR (P < 0.001; Fig. 3C).

### Blood pressure

**Fig. 4:**
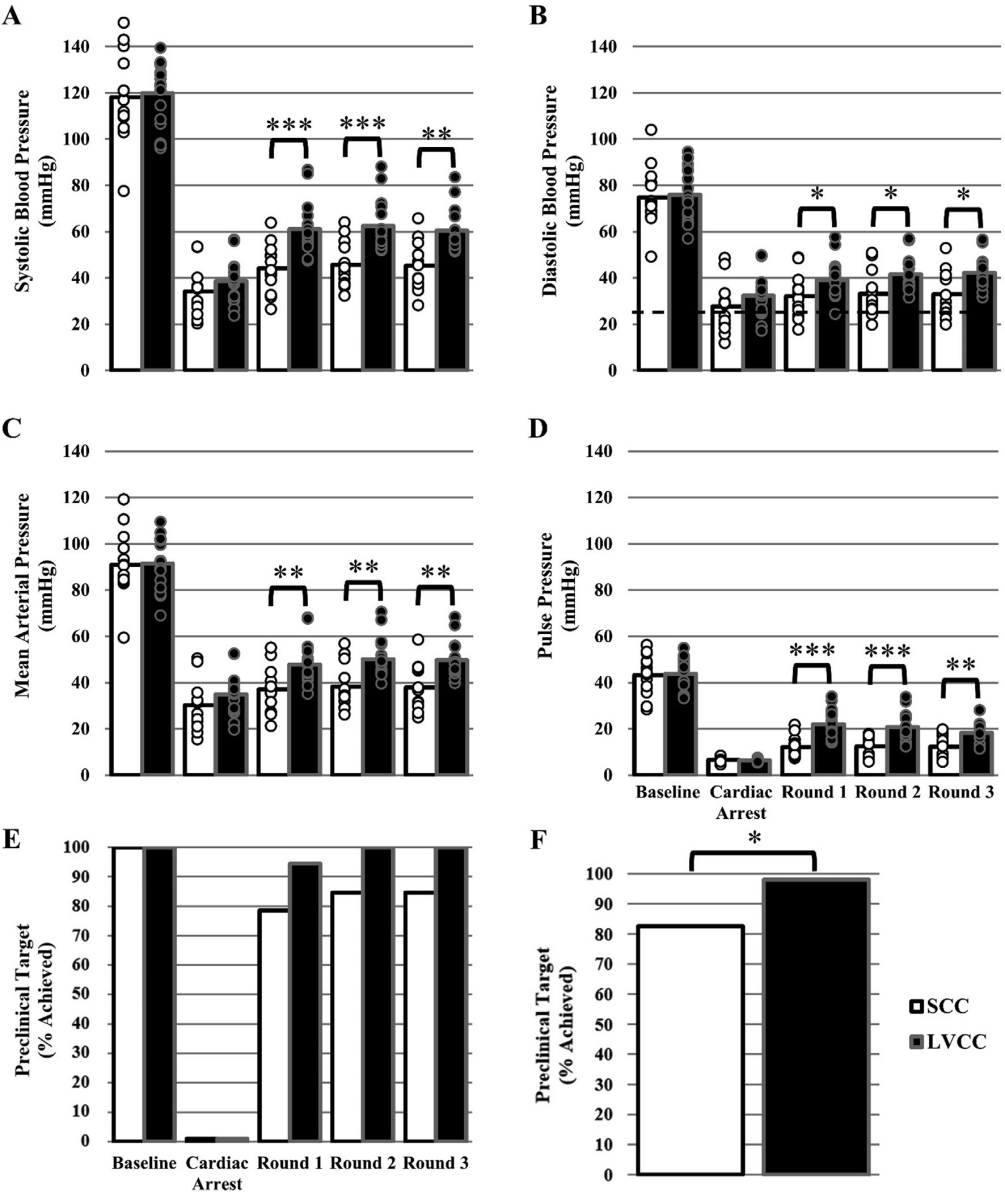
Average systolic blood pressure (SBP; Panel A), diastolic blood pressure (DBP; Panel B), mean arterial blood pressure (MAP; Panel C) and pulse pressure (PP; Panel D) values for standard chest compression (SCC; white fill bars) and left ventricle chest compression (LVCC; black fill bars) groups during baseline, cardiac arrest (CA), basic life support (BLS) cardiopulmonary resuscitation (CPR) round 1 (SCC n = 14, LVCC n = 18), CPR round 2 (SCC n = 13, LVCC n = 16), and CPR round 3 (SCC n = 13, LVCC n = 15). Data were analyzed using a mixed model ANOVA. *A priori* between group comparisons at baseline, CA, and round 1, round 2 and round 3 of BLS CPR were completed using a post hoc Tukey’s test. Significantly greater than SCC *(P < 0.05), **(P < 0.01), ***(P < 0.001). Bars represent mean. Individual data points reflect individual animals. Some individual datapoints may overlap, thus obscuring visualization. The sample sizes listed herein are accurate. Dashed black line represent preclinical target for DBP (≥25 mmHg). Percent of animals that achieved the preclinical target for DBP in the SCC and LVCC groups during baseline, CA, and round 1, round 2 and round 3 of BLS CPR (Panel E), and across all three CPR rounds cumulatively (Panel F). Panel E and F data were analyzed using Fisher’s exact tests. Significant dependency between chest compression location and achieving the preclinical target *(P < 0.05).

There was a significant group × time interaction effect (P < 0.001) for SBP values. Pairwise comparisons reveal groups were similar at baseline and during untreated CA (P ≥ 0.277), but greater in the LVCC versus SCC group in all three rounds of CPR (P ≤ 0.003; Fig. 4A). There was a main effect of group (P = 0.049) and time (P < 0.001), but the group × time interaction effect for DBP values was not significant (P = 0.078). Given there were two main effects, and in line with the stated *a priori* comparisons, between group comparisons were made at each time point. Pairwise comparisons reveal DBP was similar at baseline and during untreated CA (P ≥ 0.188), but greater in the LVCC versus SCC group in all three rounds of CPR (P ≤ 0.040; Fig. 4B). There was a significant group × time interaction effect (P = 0.002) for MAP values. Pairwise comparisons reveal groups were similar at baseline and during untreated CA (P ≥ 0.190), but greater in the LVCC versus SCC group in all three rounds of CPR (P ≤ 0.007; Fig. 4C). There was a significant group × time interaction effect (P < 0.001) for pulse pressure values. Pairwise comparisons reveal groups were similar at baseline and during untreated CA (P ≥ 0.766), but greater in the LVCC versus SCC group in all three rounds of CPR (P ≤ 0.002; Fig. 4D). The effect size analysis reveals that LVCC had a small, positive effect on SBP, DBP and MAP, and a medium, positive effect on pulse pressure in all three rounds of CPR (Table 1). Achieving the preclinical target for DBP was not dependent on chest compression location during each BLS CPR round independently (P ≥ 0.179; Fig. 4E). However, a greater percentage of animals achieved the preclinical target for DBP in the LVCC versus SCC group across all rounds of CPR (P = 0.020; Fig. 4F).

## Discussion

The current study revealed that compared with the SCC location, BLS CPR performed in the LVCC location resulted in significantly higher values for indices of systemic and cerebral perfusion. Further, the total number of rounds in which AHA targets for ETCO_2_ and DBP were achieved was greater during LVCC versus SCC. Concerning potential risks of LVCC, no group differences in prevalence of CPR-related injury were observed. Overall, the data provide evidence that, compared to SCC, LVCC results in enhanced hemodynamic status and cerebral blood flow in a swine model of BLS CPR.

In the present study, both systolic CBV and mean CBV were greater during LVCC compared to SCC. This was likely the result of increased stroke volume and potential redistribution of blood flow to the brain during compression systole. Both ETCO_2_ (accepted surrogate for cardiac output in CPR^18^, ^19^) and pulse pressure (surrogate for stroke volume^42^) were greater in the LVCC versus SCC condition, suggesting stroke volume increased with LVCC. Potentially, LVCC does not cause the same narrowing of the left ventricular outflow tract as SCC (up to 83 %),^14^, ^17^ facilitating a larger stroke volume per compression. The present study is in agreement with clinical data where transition from SCC to LVCC following 30 mins of conventional CPR produced improvements in ETCO_2_.^18^ Mechanistically, with the descending aorta being located posterior to the heart, indirect aortic compression at the level of the left ventricle may cause an advantageous aortic ballooning effect that directs an increased portion of stroke volume cranially.^17^ Indeed, previous swine work reported improved ETCO_2_ and non-significant increases in cerebral oxygenation with LVCC versus SCC (estimated effect size: ∼0.3).^19^ In view of the present results, LVCC potentially promotes superior systemic hemodynamics and cerebral perfusion compared with SCC.

Given the increased cardiac output, it follows that there would be a concomitant increase in BP. Improvements in BP with LVCC versus SCC were in concordance with previous swine work using a model of ventricular fibrillation arrest.^19^ However, despite increased BP with LVCC, no differences in diastolic CBV were observed. This may be explained by impaired autoregulation in the post-arrest hypoxic brain^43^ or BP during CPR decompression (compression diastole) being below the autoregulatory threshold of adequate cerebral perfusion,^44^, ^45^ either resulting in minimal or absent observed diastolic flow. Given increases in CBV yield from the compression phase, these data highlight the importance of high-quality compressions in minimizing cerebral perfusion deficits.

In the present study, the AHA target for ETCO_2_ (≥20 mmHg) was reached in merely 52 % of SCC versus 100 % of LVCC rounds. The AHA target for DBP (≥25 mmHg) was reached in 82 % of SCC versus 98 % of LVCC rounds throughout BLS CPR. AHA targets were met more routinely with LVCC, raising the possibility that LVCC improves the effectiveness of CPR in real-world settings.

In the current study, although more swine achieved ROSC in the LVCC (n = 3) versus SCC group (n = 0), the prevalence of ROSC was not different between groups. However, the current BLS-only study was likely underpowered to assess differences in this secondary outcome. Anderson and colleagues reported ROSC in 69 % of LVCC versus 0 % of SCC swine, and all during advanced life support CPR.^19^ Ergo, LVCC may improve cerebral blood flow and potentiate the prevalence of ROSC when epinephrine, defibrillation, or both are administered. Given the risk for CPR-related injury was similar among groups, the results herein provide a rationale for future investigations to examine the efficacy of LVCC for increasing the prevalence of ROSC in out-of-hospital CA. Indeed, our group published work suggesting that localizing the heart in CA is possible using a facile ultrasound device with artificial intelligence.^28^ Optimized LVCC may be achievable in seconds with a novel and simple semi-automated device in the out-of-hospital setting without the current need for expertise and impractical equipment.^28^

## Limitations

Data from swine models of resuscitation must be interpreted cautiously and confirmed in the clinical setting. Compression of the heart and underlying structures may differ between species. Underlying structures during CPR were not tracked and may have shifted. This was not a randomized control trial, and after the first animal was randomized, subsequent animals were assigned using a systematic rotation, alteration protocol. This approach was employed to obtain a sufficient number of complete data sets in both groups, using the fewest number of animals.^40^ An advantage of TCD is that it can be used in both preclinical and clinical settings.^24^, ^27^, ^34^, ^46^ However, TCD quantifies blood velocity and not volumetric flow. Furthermore, ETCO_2_ is a surrogate for cardiac output. Thus, more invasive preclinical work and human trials are requisite to confirm these findings.

## Conclusion

The present study provides novel insight into the benefits of LVCC versus SCC on hemodynamic performance, demonstrating that LVCC improves systemic and cerebral hemodynamic status in a swine model of asphyxiated CA and CPR. Given the observed benefits of LVCC and vitality of cerebral preservation to ROSC and survival, further studies are warranted to determine the translational relevance of these discoveries.
